# Surgical Treatment for an Infectious Aneurysm Caused by Perclose ProGlide®: A Case Study

**DOI:** 10.7759/cureus.107060

**Published:** 2026-04-14

**Authors:** Yasutaka Imamura, Hiroshi Sasajima, Kazuho Kamishima, Kazuhito Suzuki

**Affiliations:** 1 Department of Cardiology, Kugayama Heart Clinic, Tokyo, JPN; 2 Department of Cardiovascular Surgery, Kosei Hospital, Tokyo, JPN; 3 Department of Cardiology, Kosei Hospital, Tokyo, JPN

**Keywords:** coronary intervention, dialysis patient, femoral access complication, infectious pseudoaneurysm, perclose proglide, septic shock, staphylococcus aureus, vascular closure device

## Abstract

In recent years, there has been a shift from the inguinal approach to coronary artery treatment to the radial approach, which has fewer complications. However, the inguinal approach is still often used in cases of dialysis. Following inguinal puncture, vascular closure devices (VCDs) such as ANGIO-SEAL® (Terumo, Tokyo, Japan), EXOSEAL® (Hialeah, Miami Lakes, FL), and Perclose ProGlide® (Abbott Vascular Device, Santa Clara, CA) are used according to their various characteristics.

In this report, a 61-year-old male patient supported by maintenance dialysis underwent a medical examination, whereupon an abnormal electrocardiogram was produced. When a catheter was then implemented, three-vessel disease was found, and coronary artery bypass grafting (CABG) was recommended. However, upon the patient’s request, percutaneous coronary transluminal angioplasty (PTCA) was instead performed.

The patient underwent coronary artery treatment across three sessions; in the third session, an approach from the right inguinal region was taken, and Perclose ProGlide® was used for vascular closure following treatment. However, paralysis of the right lower limb occurred four days later, and the patient returned to our department, where he was found to have high inflammation levels, methicillin-susceptible *Staphylococcus aureus *(MSSA) in a blood culture, and a pseudoaneurysm in the right inguinal region as indicated by a computed tomography (CT) scan. Thus, the patient was diagnosed with an infectious pseudoaneurysm and transferred to another hospital for surgical treatment. Here, we report on a case of an infectious pseudoaneurysm that required surgery with a vascular closure device.

## Introduction

All vascular closure devices (VCDs) that may be used following inguinal puncture catheters in Japan are either collagen-based (ANGIO-SEAL® (Terumo, Tokyo, Japan) and EXOSEAL® (Hialeah, Miami Lakes, FL)) or suture-mediated devices (Perclose ProGlide® (Abbott Vascular Device, Santa Clara, CA)). Of these, only Perclose ProGlide® can be used for dialysis patients. The Perclose ProGlide® is a suture-mediated vascular closure device that achieves hemostasis by delivering a pre-tied polypropylene knot to the arteriotomy site. Unlike plug-based or collagen-based devices, it provides a “pre-close” or “primary” mechanical closure of the vessel wall, which is particularly advantageous in large-bore access management. As a suture-type device, it carries with it a low risk of blood clots and has high safety [1]. This device has also been shown to have a shorter procedure time and shorter hospital stay compared to compression vascular closure devices [2,3]. Moreover, Perclose ProGlide® also has a low reported rate of complications, with pseudoaneurysms and local infections occurring at rates of approximately 0.3% and 0.3%, respectively [4]. While clinical trials have cited a low complication rate of approximately 0.3% for both pseudoaneurysms and local infections, it is crucial to note that these figures primarily reflect the general population. In the dialysis cohort, these risks are likely underestimated, as end-stage renal disease significantly heightens the susceptibility to vascular failure and microbial colonization. There have been reports of infections and infectious aneurysms in the hemostasis area after hemostasis with Perclose ProGlide® as case reports, but the number is small. Herein, we report a rare case of an infectious aneurysm following Perclose ProGlide® use that required surgical treatment and resulted in a fatal outcome. Infectious pseudoaneurysms, although rare, represent a grave clinical entity characterized by high rates of vascular rupture, sepsis, and limb loss. Unlike sterile pseudoaneurysms caused by mechanical failure alone, infectious variants involve microbial invasion of the arterial wall, leading to rapid tissue destruction. Their clinical significance lies in their subtle early presentation, which often masks a life-threatening progression, necessitating prompt diagnosis and complex surgical or multidisciplinary intervention.

## Case presentation

The patient was a 61-year-old man with a body mass index of 25.4, hypertension, and a previous history of diabetes under oral treatment. Hemodialysis had been introduced at the age of 52 due to chronic glomerulonephritis. Electrocardiogram changes were observed at the age of 60, and adenosine-loaded myocardial scintigraphy showed a summed stress score of 18 (26.47%). Significant myocardial ischemia was observed, and thus, a catheterization study was conducted. The procedure was performed via a right femoral artery approach. Coronary angiography results indicated three-vessel disease with #1-3 75%, #3-4PD 99%, #AV 100%, #6-7 75%, #9-1 90%, #9-2 75%, #13 90%, and #14 75%. The syntax score was 41, and coronary artery bypass grafting was recommended. Despite a Syntax score of 41, percutaneous coronary intervention (PCI) was selected over coronary artery bypass grafting (CABG) because the patient was considered a high-risk surgical candidate due to end-stage renal disease and frailty, and catheter treatment was conducted instead. It was decided that the treatment was to be conducted over three sessions.

The first treatment was for the anterior descending branch using a right inguinal puncture, and a 6-French (Fr) sheath was used. The procedure time was 1 hour and 17 minutes, and vascular closure was conducted with Perclose ProGlide®. Treatment for the circumflex branch was conducted one month later. A left inguinal puncture was created with a 6-Fr sheath. Vascular closure was conducted with Perclose ProGlide®, and the procedure time was one hour. One and a half months later, treatment for the right coronary artery was conducted. A right inguinal puncture was created with a 6-Fr sheath. The guide catheter IL 3.5 was used initially, but the backup was poor, and it was changed to AL 1.5. Severe calcification of the lesion was also observed, and a Rotablator (Boston Scientific, Natick, MA) was then used. The procedure was ultimately successful, but its duration was longer than typical at 3 hours and 20 minutes due to the difficulty. Perclose ProGlide® was used for vascular closure.

**Figure 1:**
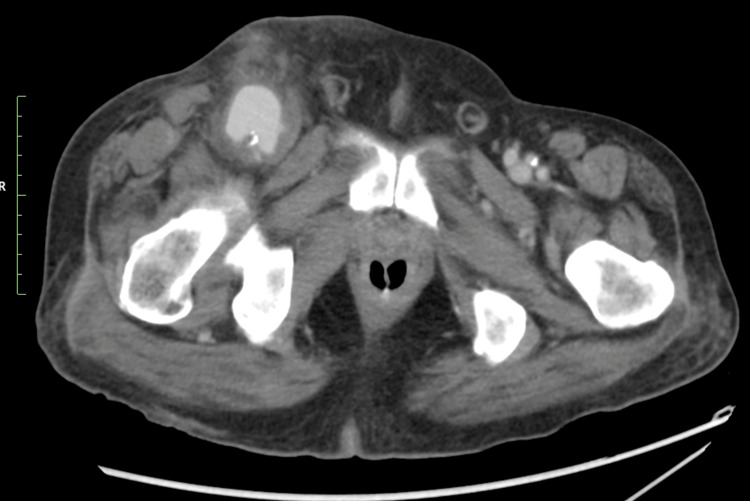
Contrast-enhanced computed tomography image of a pseudo arterial aneurysm Contrast-enhanced computed tomography demonstrating a pseudoaneurysm in the right inguinal region (approximately 46 × 41 mm), with surrounding inflammatory changes.

**Figure 2:**
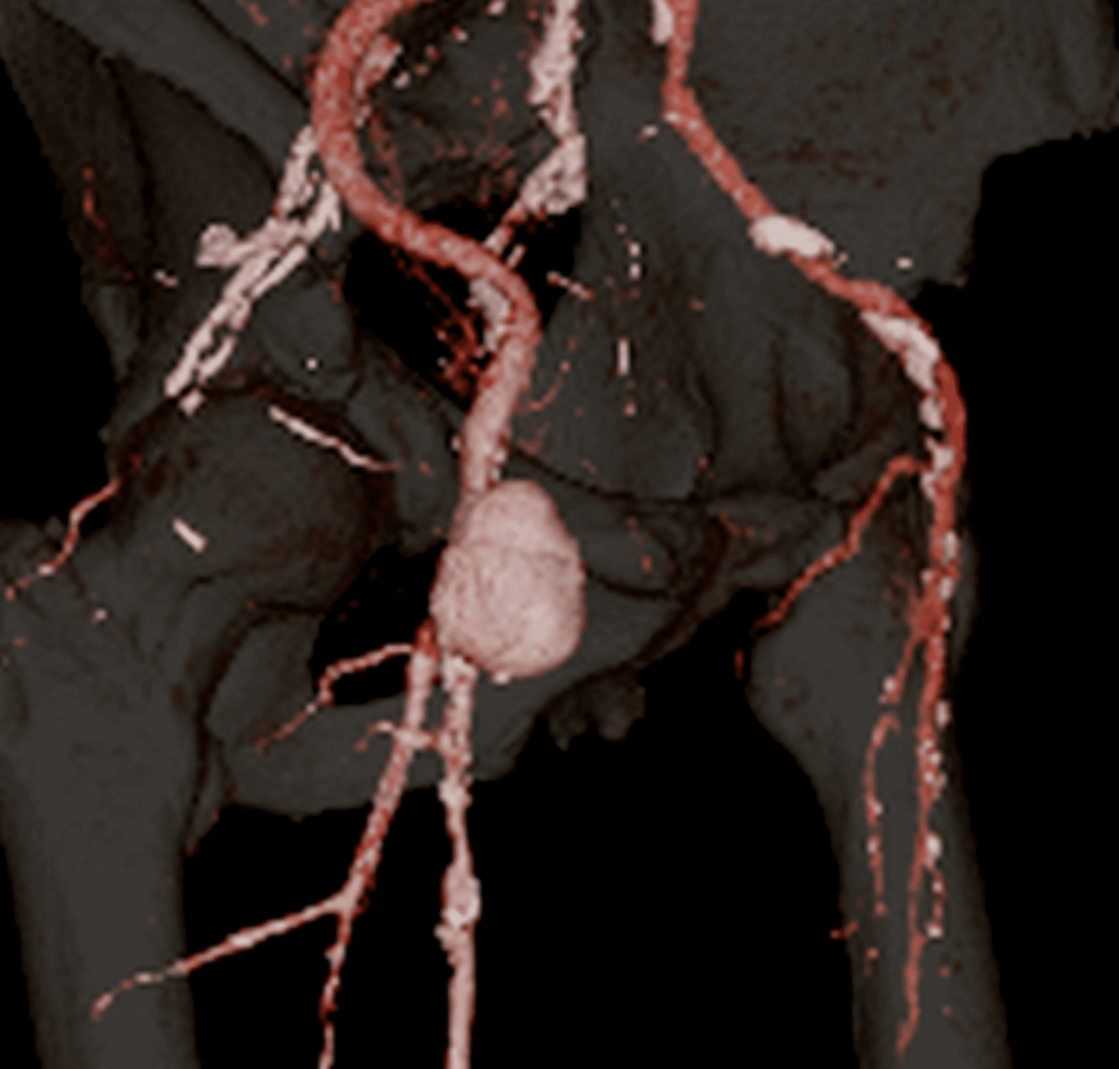
Three-dimensional reconstructed computed tomography image showing the morphology and spatial extent of the pseudoaneurysm arising from the right femoral artery

The patient was discharged from the hospital the next day in the absence of any bleeding or hematoma from the postoperative wound. However, the patient was admitted to our hospital five days later with difficulty moving his right lower limb. Contrast-enhanced computed tomography (CT) scans revealed a 46 × 41 mm pseudoaneurysm in the right inguinal region (Figure 1 and Figure 2), and the patient was admitted to the hospital for treatment.

**Figure 3:**
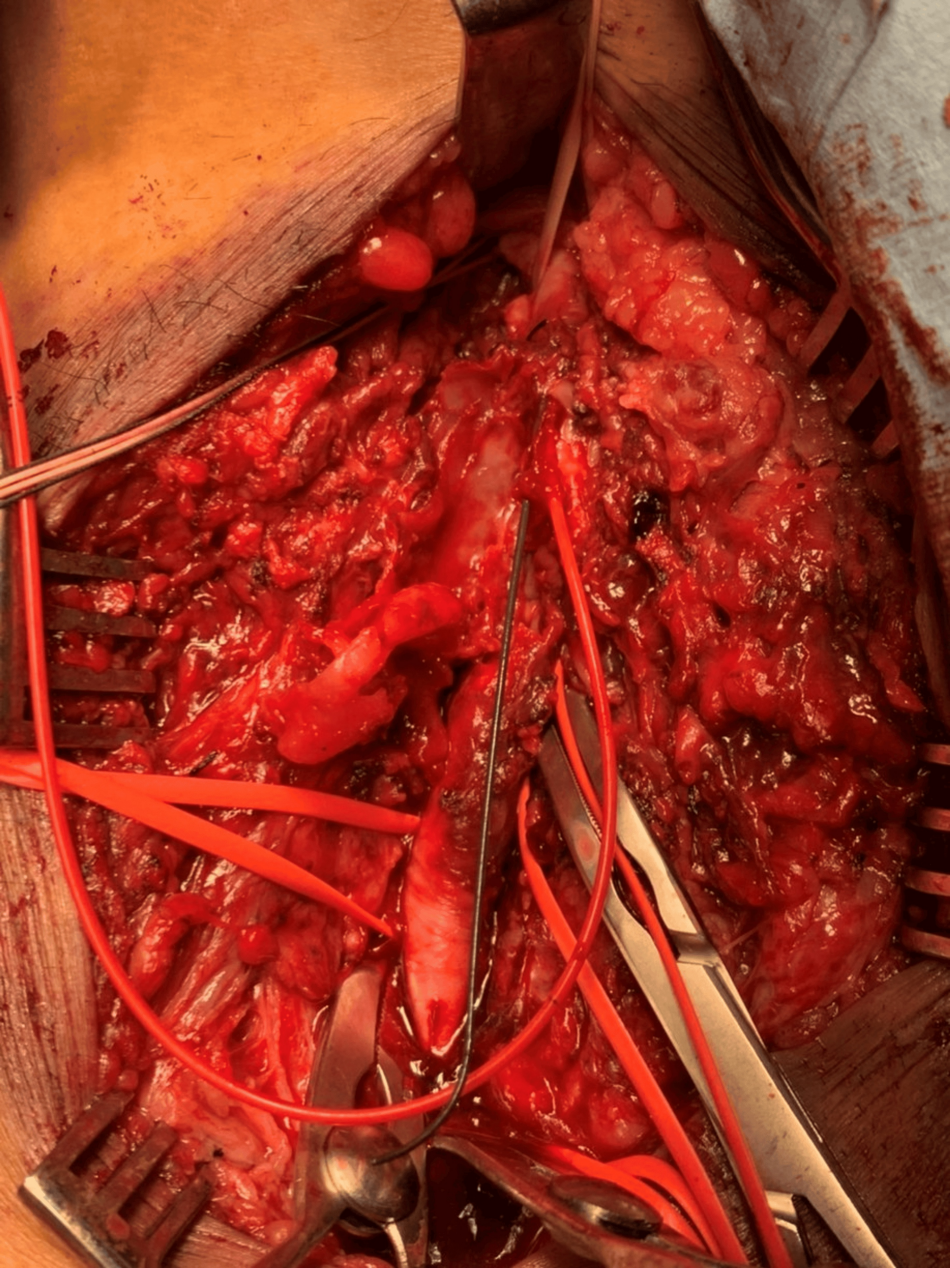
Intraoperative photo of infectious pseudoaneurysm removal Intraoperative findings during surgical removal of the infectious pseudoaneurysm. The infected aneurysmal sac was resected, followed by vascular reconstruction using a venous graft.

Blood collection at that time showed a high C-reactive protein value of 40 mg/dL, and thus, a blood culture was conducted. The next day, the blood culture was positive for gram-positive cocci in four of four sets, and the diagnosis was updated as an infectious pseudoaneurysm. Surgery was conducted on the same day by the vascular surgery department. The gram-positive cocci were later identified as methicillin-susceptible *Staphylococcus aureus*. The femoral aneurysm was removed, and a venous graft was interposed (Figure 3).

However, one month later, hemorrhagic shock occurred from the vein, and bypass surgery was conducted using the great saphenous vein. The patient suffered from non-occlusive mesenteric ischemia (NOMI) during that time, and the small intestine was resected. The patient was then stable, although in shock due to infection and acidemia owing to NOMI in the large intestine. Colorectal resection was conducted again for NOMI, but septic shock continued to persist. Despite the administration of antibacterial and pressor agents together, the patient exhibited poor improvement. Despite emergency surgical resection of the aneurysm and targeted antibiotic therapy, the clinical course was complicated by persistent sepsis, disseminated intravascular coagulation (DIC), and progressive multi-organ failure. Following a four-week interval of intensive care after the diagnosis of the pseudoaneurysm, the patient ultimately succumbed to septic shock. This fatal outcome occurred approximately four months after the initial right coronary artery procedure.

Key clinical features for identification

To facilitate early identification of similar presentations, the following key markers observed in this case should be highlighted: persistent fever in a patient with a history of VCD use, even months after the procedure; neurological symptoms (e.g., limb paralysis) caused by the mass effect of a rapidly enlarging pseudoaneurysm; and high susceptibility in hemodialysis patients with extensive arterial calcification, where standard hemostasis may be compromised.

## Discussion

This study shared the case of a patient with a high risk of infection who underwent catheter treatment by inguinal puncture and vascular closure with Perclose ProGlide®. The development of the infectious pseudoaneurysm in this case appears to be the result of a complex interplay between the patient’s systemic vulnerabilities and technical challenges encountered during the procedure. Specifically, we believe that the combination of severe arterial calcification, prolonged sheath manipulation, and the immunosuppressive state of end-stage renal disease created a “perfect storm” for bacterial colonization at the VCD deployment site.

Patients at high risk for general infection include diabetic patients, dialysis patients, patients who have undergone long-term procedures, patients who have undergone multiple sheath and device changes, patients who have had sheaths inserted for extended periods of time, patients who have undergone ipsilateral inguinal access within two weeks, obese patients with pleated skin, immunocompromised patients, patients with artificial implanted valves or significant valve lesions, patients with artificial joints, patients who have undergone long-term hospitalization, patients with poor hygiene, patients with co-existing infections in distant body parts, patients with implanted thigh grafts, and patients in home care or nursing homes [5-8]. In the present case, the patient was a diabetic and used dialysis, and the third catheter treatment encountered difficulties requiring guide catheter replacement and long-term sheath insertion. Additionally, although not within the preceding two weeks, the inguinal region on the same side had been used as an access site two and a half months previously. In retrospect, the patient had a sufficiently high risk of infection.

Furthermore, caution should be exercised when establishing a direct causal link solely to the VCD, as anatomical and systemic factors significantly increased the patient’s predisposition to complications. CT angiography revealed extensive arterial calcification, which necessitated the use of larger sheaths for complex lesions; this combination inherently complicates the achievement of a secure arterial seal with suture-mediated devices. Additionally, the role of hemodialysis extends beyond general immunosuppression; it creates a clinical setting that facilitates bacterial entry, with *Staphylococcus *species, the pathogens identified in this case, being among the most frequent culprits in this population. Indeed, the clinical literature supports a significantly elevated risk in this cohort; for instance, patients on maintenance hemodialysis have been reported to have a several-fold higher relative risk of surgical site and vascular access infections compared to non-dialysis patients. While the complication rate for pseudoaneurysms and infections is often cited as approximately 0.3% in the general population, this figure likely represents a substantial underestimation for dialysis patients, whose hazard ratio for major vascular complications is markedly increased due to impaired vascular integrity and systemic immunosuppression. Compared to previous literature regarding VCD-related infections, which typically manifest within 2-4 weeks, our case is unique for its delayed presentation (2.5 months post-procedure) and its neurological manifestation (limb paralysis). While most infectious pseudoaneurysms present with sepsis or local hemorrhage, the late enlargement of the aneurysm in this dialysis patient led to a significant mass effect on the femoral nerve. This suggests that in high-risk patients with calcified vessels, clinicians should maintain a high index of suspicion for late-onset complications, even months after a seemingly successful closure.

Device-related infections have a low incidence and thus have not undergone randomized prospective trials. Furthermore, routine prophylactic antibiotics during cardiac catheterization are currently not recommended, as the question of whether or not they are necessary remains unanswered [6]. In this case, the inguinal region was shaved and disinfected in a concentric manner from the center to the outer edge using 10% povidone iodine prior to treatment, showing that even when maximal barrier precautions are conducted, an infectious aneurysm can develop; thus, prophylactic administration of antibiotics may also be considered when using vascular closure devices in patients with multiple risks of infection. It has also been reported that chlorohexidine has favorable persistence on the skin and shows a better skin disinfecting effect when combined with alcohol. Therefore, use of chlorhexidine alcohol with a concentration exceeding 0.5% is recommended over povidone iodine in order to reduce catheter colonization [6,9,10]. Therefore, it seems that chlorhexidine should be used at a concentration greater than 0.5% for preoperative disinfection. At present, the best course of action must be determined according to each individual patient, and additional research is required to arrive at a uniform decision.

## Conclusions

In the present case, a patient with a high risk of infection developed a fatal infectious pseudoaneurysm following catheter treatment and vascular closure with Perclose ProGlide®. While our findings are derived from a single clinical observation, this case illustrates that an infectious pseudoaneurysm can develop even with maximal barrier precautions in high-risk patients. Although routine prophylactic antibiotics for cardiac catheterization are not currently established as a standard of care, our experience suggests that their administration might be considered on an individual basis for patients with multiple risk factors, such as end-stage renal disease and severe arterial calcification. Ultimately, clinicians must maintain high vigilance for post-procedural infection in such vulnerable populations, and further large-scale studies are warranted to determine the definitive efficacy of prophylactic protocols in these settings.
